# Comparing of two different epidemic seasons of bronchiolitis

**DOI:** 10.1186/s13052-018-0454-4

**Published:** 2018-01-16

**Authors:** Simonetta Picone, Adele Fabiano, Davide Roma, Piermichele Paolillo

**Affiliations:** NICU Casilino General Hospital, Rome, Italy

**Keywords:** Bronchiolitis, Prophylaxis, Preterm, Palivizumab

## Abstract

Acute bronchiolitis is the most common cause of hospitalizations in infants < 12 months of age and preventive efforts remain the most important strategy to date. Recently prophylaxis with palivizumab (PLV) was limited to preterm infants with < 29 weeks gestational age (wGA).

We performed a single center analysis in preterm infants (GA between 30 and 32 weeks) and age < 12 months to compare prophylaxis with PLV and frequency and characteristics of bronchiolitis and bronchiolitis-related hospitalization in two consecutive epidemic seasons (S1 vs S2).

We found a rising trend in rate of bronchiolitis and bronchiolitis-related hospitalization in S1 vs S2. Among hospitalization, we found an increased morbidity with an increase in the rate of mechanical ventilation in S2. Additionally, hospitalization occurred in subjects with younger chronological age in S2 compared with S1.

Our result cannot be generalized because deriving from a single Center and further evaluation on wider simple size are warranted, but it suggests an increase in the incidence, gravity and precocity of bronchiolitis in 29–32 wGE preterm infants with the change in National guidelines for prophylaxis.

## Dear editor,

It is well known that acute bronchiolitis is the most common cause of hospitalizations in infants < 12 months [1] of age and to date no specific therapy was found to be effective for the treatment of the disease in this patient population [2]. Thus, environmental and pharmacological prevention is considered a public health challenge and the prophylaxis with palivizumab (PLV, a monoclonal antibody produced by recombinant DNA technology) is indicated during the epidemic period to prevent serious respiratory syncytial virus (RSV) infections and associated complications in children at risk. Prematurity is one of the most important risk factors for severe RSV infection. In 2014 the American Accademy of Pediatrics (AAP) removed the newborn with > 29 week of gestational age from the hight risk group to receive PLV prophylaxis [3]. In October 2016 the Italian Drug Agency (AIFA) modified the indication for the financial coverage limited it only for newborn with < 30 week of gestational age [4]. Until this date, prophylaxis was offered to all newborn with a < 35 week of gestational age and with less of 6 months at the start of epidemic seasons that in Italy goes from October to April.

We performed a single Center, retrospective analysis in preterm infants with GA ranging from 30 (30 + 0) to 32 (32 + 6) weeks and age < 12 months. The aims of the analysis were to evaluate the difference in the frequency of bronchiolitis and in the frequency and characteristics of the bronchiolitis-related hospitalization between the two seasons. Clinical information on outcomes were gathered by phone interviews with parents at the end of June 2017 for the two groups and relevant information surrounding outcomes were extracted from electronic hospital records. Infants born in our Center from 1st of May 2015 to 31st of March 2016 (analyzed for epidemic season 2015–2016) and from 1st of May 2016 to 31st of March 2017 (analyzed for epidemic season 2016–2017) and belonging to the gestational age group of 30–32 weeks were included in the analysis. As alluded to before, collected data included the presence/absence of pharmacological prophylaxis, diagnosis of bronchiolitis and bronchiolitis treatment, mechanical ventilation. Data regarding respiratory assistance during neonatal hospitalization were also collected as reported in Table 1.

**Table 1:** Clinical characteristic of two groups (WGE = week of gestational age)

	S1 (2015–2016)	S2 (2016–2017)	*P* value
Newborn with WGE 30^+ 0^–32^+ 6^	35	47	
Mean gestational age	30,9	31	
Newborn with WGE < 31 sett (30^+ 0^–30^+ 6^)	16/35 (45,7%)	18/47 (38,3%)	*p* = 0,5
Newborn treated with PLV	27	2	*p* < 0,05
Number of bronchiolitis	6/35 (17%)	12/47 (26%)	*P* = 0,36
Recovered for bronchiolitis	3/6 (50%)	6/12 (50%)	*p* = 1
Intubation at birth	3/3 (100%)	4/6 (66,7%)	*P* = 0.5
Age at the recovery (Months)	7	4.3	
Intubation at recovery	1/3 (33,3%)	2/6 (33,3%)	*p* = 1

Newborns with GA ranging from 30 to 32 weeks were 47 for the 2015–16 epidemic season (S1) and 64 for the 2016–17 season (S2). 29 infants were excluded from the principal analysis (12 from S1 and 17 from S2 because of missing information). According to the AIFA recommendations for 2015, during the 2015–2016 epidemic period, 27 infants out of 35 (77%) underwent PLV prophylaxis, a proportion which was more than 18 times higher than that observed during the subsequent (2016–2017) epidemic period (2/47,4,2%).

The percentage of bronchiolitis raised from 17% (6/35) in S1 to 26% (12/47) in S2. Of note, at variance with the previous season (when no invasive ventilation was required), during the 2016–2017 epidemic period, 33% of hospital admissions needed intubation.

The mean and median chronological age at hospitalization for bronchiolitis were > 6 months in S1 (mean and median value =7 months), with one child hospitalized for bronchiolitis at 5th and 9th months after births. In S2, the mean and median age at hospitalization for bronchiolitis were < 6 months (mean = 4.3 months, median = 3.0 months) with 83% of admissions for bronchiolitis occurring at < 6 months of chronological age, with 66% in the first 3 months of life.

In S1 we recorded only two hospitalizations occurred in one patient who was critically instable at births, on the contrary 33,3% (2/6) of hospitalizations for bronchiolitis occurred in children not requiring respiratory assistance at births in S2**.**

We have found a rising trend in rate of bronchiolitis and bronchiolitis requiring hospitalization from S1 to S2. Among hospitalized bronchiolitis, we found an increased morbidity as suggested by the need of intubation procedures in an high proportion of hospitalizations during S2. Additionally, bronchiolitis and hospitalization occurred in subjects with younger chronological age in S2 compared with S1. Statistical analysis of the data didn’t show any significant difference between the two group probably because of the small number of samples being analyzed.

Our result cannot be generalized because deriving from a single Center and further evaluation on wider simple size are warranted, but it suggests an increase in the incidence, gravity and precocity of bronchiolitis in 29–32 wGE preterm infants with the change in National guidelines for prophylaxis.

## References

[1] Nair H, Nokes DJ, Gessner (2010). Global burden of acute lower respiratory infections due to respiratory syncytial virus in young children: a systematic review and meta-analysis. Lancet.

[2] Baraldi E, Lanari M, Manzoni P (2014). Inter-society consensus document on treatment and prevention of bronchiolitis in newborns and infants. Ital J Pediatr.

[3] Committee on infectious diseases and bronchiolitis Guidelines Committee (2014). Updated guidance for palivizumab prophylaxis among infants and young children at increased risk of hospitalization for respiratory syncytial virus infection. Pediatrics.

[4] Gazzetta Ufficiale della Repubblica Italiana. GU Serie Generale n.221 del 21–9 2016. http://www.gazzettaufficiale.it/eli/gu/2016/09/21/221/sg/pdf. Accessed 21 Sept 2016.

